# Effect of patient-controlled intravenous analgesia combined with flurbiprofen axetil and dezocine on postoperative analgesia for lobectomy (EPIC-FAD): a trial protocol

**DOI:** 10.1186/s13063-021-05108-9

**Published:** 2021-03-01

**Authors:** Jian Zhou, Qiang Pu, Lin Lin, Weelic Chong, Boran Chen, Yang Hai, Fei Liu, Lunxu Liu

**Affiliations:** 1 Department of Thoracic Surgery, West China Hospital, Sichuan University, Chengdu, No. 37, Guoxue Alley, Chengdu, 610041 Sichuan China; 2 West China School of Medicine, Sichuan University, Chengdu, China; 3 Sidney Kimmel School of Medicine, Thomas Jefferson University, Philadelphia, PA USA; 4 Department of Anesthesiology, West China Hospital, Sichuan University, Chengdu, China; 5 Western China Collaborative Innovation Center for Early Diagnosis and Multidisciplinary Therapy of Lung Cancer, Sichuan University, Chengdu, China

**Keywords:** Postoperative analgesia, Lobectomy, Patient-controlled intravenous analgesia flurbiprofen axetil, Dezocine

## Abstract

**Background:**

The optimal analgesic strategy for surgical pain after lobectomy remains undefined. To compare the combination of flurbiprofen axetil and dezocine with flurbiprofen axetil alone and dezocine alone, in post-lobectomy patients.

**Methods:**

A single-center, parallel-design double-blind superiority trial, with 5 groups (1:1:1:1:1 ratio) with different combinations of flurbiprofen and dezocine. Patients scheduled for lobectomy will be recruited. The primary outcome is total sufentanil use in patient-controlled intravenous analgesia within the first 24 postoperative hours. Secondary outcomes include pain numeric rating scales at 6th, 12th, 24th, 48th, and 72th postoperative hours, and on the 1st, 3rd, and 6th postoperative months at rest and during coughing, adverse effects from experimental drug treatment, sufentanil use at other time points, analgesia cost, time to chest tube removal, length of hospital stay, time to pass first flatus, and serum level of cytokines. Doctors, patients, and nurses are blinded, and only the manager is unblinded. Analysis is intention-to-treat. Statistical analysis is pre-specified. Statistical comparison of the treatment groups includes one-way analysis of variance followed by Tukey’s post hoc test.

**Discussion:**

Trial did not begin to recruit. Participant recruitment start date is planned to be June 1, 2020. Approximate recruitment end date is May 31, 2021. If successful, the trial may shed light on the use of certain analgesic combinations in post-lobectomy pain control.

**Trial registration:**

Chinese Clinical Trial Registry ChiCTR1800018563. Registered on September 25, 2018.

**Supplementary Information:**

The online version contains supplementary material available at 10.1186/s13063-021-05108-9.

## Background

Postoperative pain is one of the most common complications after surgery [1]. As observed in the US population, 86% of patients experienced postoperative pain and 74% persisted after discharged [2]. Less than half of the patients reported adequate pain relief, which suggests that management is far from sufficient [3]. Specifically, postoperative pain adversely leads to insufficient coughing, immobility, and malnutrition among patients with lobectomy, which could result in prolonged functional recovery [4, 5].

Various perioperative interventions and management strategies have been developed to manage postoperative pain [6]. These include (1) patient-controlled intravenous analgesia (PCIA), which enables patient-oriented drug use [7]; (2) conventional intravenous injection [8]; (3) oral analgesic medications [9]; (4) epidural analgesia [10]; (5) paravertebral blockade [11]; (6) surgical wound infiltration [12]; (7) transdermal therapeutic system [13], etc. Opioids are among the mostly prescribed medications, whereas non-steroidal anti-inflammatory drugs (NSAIDs) are usually recommended for multimodal analgesic therapy [14, 15]. Over the past few years, the combined use of different analgesics for postoperative pain management has increasingly attracted the attention of surgeons and researchers [16]. In China, dezocine is one of the most widely used opioids for postoperative pain relief, far superseding use of morphine for pain control [17]. Wu et al. conducted a randomized controlled trial (RCT) recruiting 60 thoracotomy patients and found that the combination of dezocine and morphine had better analgesic effects and less adverse effects over using dezocine alone [18]. Flurbiprofen axetil is one of the NSAIDs applied in this clinical setting as well [19]. As Wu et al. demonstrated in patients with cancer, opioids incorporating with flurbiprofen axetil yielded better pain relief and reducing drug consumption in comparison to opioids alone [20]. Flurbiprofen axetil and dezocine have different analgesia mechanisms. The former inhibits prostaglandins release to reduce the sensitivity of local pain receptors, while the latter acts on the κ opioid receptor in the central nerve system [17, 19]. However, the benefit of this combined regimen was not tested in pain control after lobectomy. On the basis of our preliminary clinical observations, we proposed that a combination of flurbiprofen axetil and dezocine may elicit a synergistic antinociceptive effect, which allows for reduced drug intake of each constituent drug and ultimately less adverse effects. Thus, we conduct this RCT to identify the efficacy and safety of flurbiprofen axetil combined with dezocine on pain management after lobectomy.

## Methods and analysis

### Trial design

**Fig. 1:**
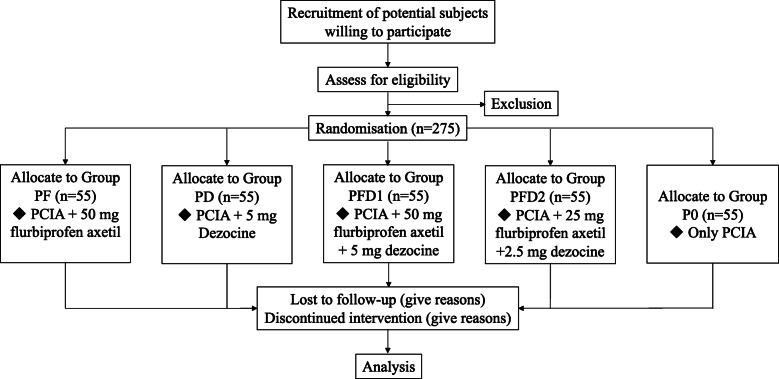
The flow chart of this study

**Fig. 2:**
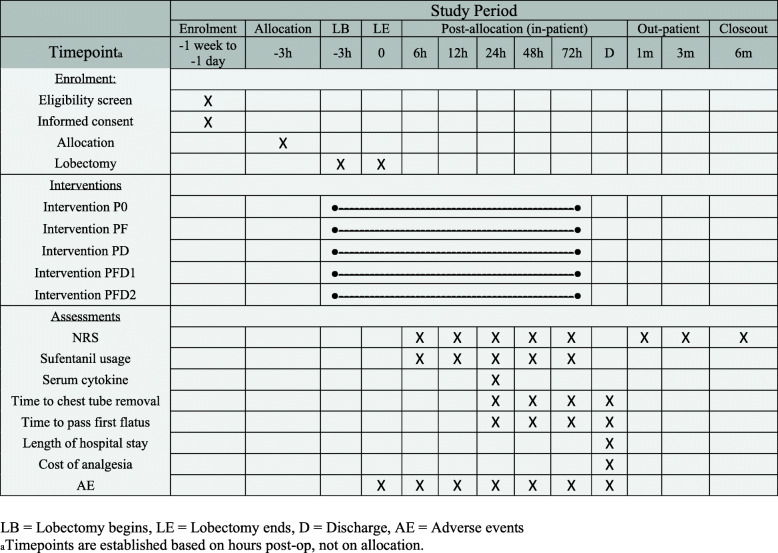
Schedule of enrolment, interventions, and assessments

This is a prospective, randomized, double-blind, parallel-controlled, and superiority trial (Fig. 1), which follows the SPIRIT reporting guideline (see Additional file 1). The time schedule of enrolment, intervention, and follow-up were summarized in the SPIRIT figure (Fig. 2).

### Study objective

The primary objective of this study is to compare the synergistic antinociceptive efficacy and the resultant decrease of adverse effects among different combinations of flurbiprofen axetil and dezocine in patients after lobectomy. The analgesia efficacy was assessed with the consumption of sufentanil in PCIA (evaluated at 6th, 12th, 24th, 48th, and 72th postoperative hours) and the score of numeric rating scales (evaluated at the 6th, 12th, 24th, 48th, and 72th postoperative hours and 1st, 3rd, and 6th postoperative months). The adverse effects were assessed by the incidence of treatment-related adverse effects.

### Study setting

This study will be conducted on patients undergoing lobectomy in the Department of Thoracic Surgery, West China Hospital, Sichuan University. This institution is a public academic hospital located in the western China, which ranked at the 1st (in China) and the 25th (all over the world) in the Nature Index 2020 Tables in Healthcare Institutions [21].

### Recruitment

#### Participants

Patients eligible for the trial must comply with all the inclusion criteria and do not meet any exclusion criterion before enrolment. To achieve adequate participant enrolment, all physicians in the thoracic department of the hospital are informed of the trial, and to contact the trial coordinator if they encounter potential eligible patients. All the included patients will sign an informed consent form, which should contain (1) detailed information of the study design, (2) benefits of participating, (3) risk of respiratory and circulatory depression, and (4) remedial action for patients who experienced any harms of the intervention. A trained individual researcher will obtain informed consent from the patient, in a separate setting from the trial coordinator.

#### Inclusion criteria

Patients who, at the time of enrolment, meet all the following criteria, are eligible for the study:

1. Patients aged between 18 and 70
2. Patients undergoing planned lobectomy
3. ASA risk class of I–II
4. Ability to sign an informed consent form
5. Ability to understand all the study procedures and communicate clearly with the researchers and staff

#### Exclusion criteria

Patients who, at the time of enrolment, meet any of the following criteria are not eligible for the study:

1. Body mass index (BMI) > 30 kg/m^2^
2. Last smoked < 2 weeks prior to surgery for current smokers
3. Pregnant or breastfeeding females (females aged 18 to 55 will receive pregnancy test)
4. Hepatic (presence of ≥ 3 times the reference value of alanine aminotransferase/aspartate aminotransferase), renal (undergoing renal replacement therapy before surgery), or cardiac (the left ventricular ejection fraction < 30%) dysfunction [22, 23]
5. History of gastrointestinal disease (e.g., peptic disease, intestinal perforation)
6. Severe or unstable mental illness (presence of mental illness in International Classification of Diseases-10 diagnosis groups F2, F3, F4, F5, or F6) [24]
7. Severe or unstable physical conditions in which the selective surgery would not be considered (e.g., cerebral stroke, cardiac ischemia, congestive heart failure, hepatic failure, grade 3 hypertension)
8. Patient with known allergies to opioids or NSAIDs
9. Current or recent (within 1 month prior to surgery) administration of opioids or NSAIDs.
10. Intake of any analgesics within 48 h prior to surgery
11. History of alcohol, opioids or other drugs abuse, or chronic use of opioids
12. Patients with contradictions to the use of PCIA
13. Preoperative pain diagnosis (e.g., chronic pain, cancer-causing pain in site of surgery)
14. Emergency surgery
15. Reoperations for severe postoperative complications (e.g., severe bleeding, bronchopleural fistula)

#### Drop-out criteria

Patients will be able to drop out at any time of this study under the following circumstance:

1. After recruitment, the condition of the patient changes, and the surgeon in charge confirms that emergency surgery is needed.
2. Patients can request a withdrawal to terminate the experimental treatment due to any personal reasons at any stage after recruitment.
3. Any protocol violations occur.

#### Randomization and blinding

We will perform the randomization by assigning candidates to 5 groups (Fig. 1) based on computer-generated random numbers shortly prior to the surgery. In order to maintain allocation concealment, the randomly-generated group name will be printed and placed in consecutively numbered, separate sealed opaque envelopes which will be only opened once a patient is deemed eligible. When receiving a patient who meets the inclusion criteria, an unblinded trial manager will assign the participant to a group according to the number in the envelope.

All the drugs will be packed with the same outlook by a research pharmacist who is blind to the allocation of patients. The investigators, treating doctors and nurses, as well as the study participants are blinded to the allocation of the participants, while the project manager remains unblinded. In case of unexpected emergency circumstances, the randomization and allocation will be disclosed to the investigator. The blinding will be discontinued after all data analyses are completed. If unblinding occurs, the participants will discontinue the study medication and a complete report will be made.

#### Sample size

We estimated the sample size based on one-way analysis of variance (ANOVA) of total consumption of sufentanil in PCIA within the first 24 postoperative hours in group PD and group P0. The estimated consumptions of sufentanil in PCIA within the first 24 postoperative hours were 63 and 71 μg in group PD and group P0, respectively [25]. Standard deviations of each group were estimated as 1.15, and effect size of 0.67 was calculated [25]. The numbers of participants will allow a 5% chance of type I error and have 95% power. The sample size of 49 in each group was calculated by using G*Power (Software Version 3.1.3, University of Düsseldorf, Germany). Based on prior experience at our center, we set the dropout rate as 10% to account for the inability to complete the treatment, data errors, loss to follow-up, and other unanticipated study problems. The adjusted sample size for each group is 55. Thus, this study needs to recruit a total of 275 participants.

#### Intervention

A total of 275 subjects who are scheduled for lobectomy will be recruited in our study according to inclusion and exclusion criteria. All patients are equally allocated into 5 groups: Group P0: only PCIA, Group PF: PCIA + flurbiprofen axetil, Group PD: PCIA + dezocine, Group PFD1: PCIA + flurbiprofen axetil+ dezocine, and Group PFD2: PCIA + flurbiprofen axetil (half-dose) + dezocine (half-dose).

All the participants will receive electronic PCIA as the basic component of postoperative pain management once awake after anesthesia. The PCIA drug formulation is 1 μg/mL of sufentanil citrate (Yichang Humanwell Pharmaceutical Co., Ltd., Yichang, China), 15 mg of tropisetron hydrochloride (Jiangsu Hengrui Medicine Co., Ltd., Jiangsu, China), and 200 μg of dexmedetomidine (Taiji group·Southwest Pharmaceutical Co., Ltd., Chongqing, China) combined with normal saline into 200 mL solution. The PCIA drug will be administered as a single 2-mL dose at a 10-min locking time, with a maximum dose of 10 mL every hour and a background input quantity of 0.5 mL/h. Other than PCIA, all drugs are administered intravenously by nurses coordinated by the unblinded manager in the trial. None of the experimental drugs are taken orally, eliminating the need to monitor adherence. All the following study analgesics, including PCIA, are discontinued at 72 h after operation. If pain cannot be controlled after 72 postoperative hours, PCIA is continued as a “rescue” analgesic strategy to achieve a pain score of numeric rating scales (NRS) ≤ 5. An extra 5 mg of dezocine [Yangtze River Pharmaceutical (Group) Co., Ltd., Yichang, China] via intramuscular injection will be administered when intolerant pain happens. The dosage of added analgesics and administration timepoints will be recorded. If the above analgesic regimens cannot relieve pain, the patient will be withdrawn from the study and allowed additional analgesic regimens which are deemed appropriate, and a complete report will be made.

#### Group P0: only PCIA

##### Group PF: PCIA + flurbiprofen axetil

In addition to PCIA, patients will receive 50 mg flurbiprofen axetil via intravenous injection (Beijing Tide Pharmaceutical Co., Ltd., Beijing, China) when surgery begins as well as on the evening of the day of operation. From the postoperative day (POD) 1, the dosage will be given every 12 h.

##### Group PD: PCIA+ dezocine

In addition to PCIA, 5 mg dezocine (Yangtze River Pharmaceutical (Group) Co., Ltd., Yichang, China) will be intravenous injected when surgery begins as well as on the evening of the day of operation. From POD1, dosage will be given every 12 h.

##### Group PFD1: PCIA + flurbiprofen axetil + dezocine

In addition to PCIA, patients will receive 50 mg flurbiprofen axetil combined with 5 mg dezocine via intravenous injection when surgery begins as well as on the evening of the day of operation. From POD1, the dosage will be given every 12 h.

##### Group PFD2: PCIA + flurbiprofen axetil (half-dose) + dezocine (half-dose)

In addition to PCIA, patients will receive of 25 mg flurbiprofen axetil combined with 2.5 mg dezocine via intravenous injection when surgery begins as well as on the evening of the day of operation. From POD1, the dosage will be given every 12 h.

#### Anesthesia and operations

All operations will be performed under general anesthesia with double-lumen tracheal intubation. Five-lead electrocardiogram (ECG), pulse oxygen saturation (SpO_2_), bispectral index (BIS), end-tidal carbon dioxide tension (EtCO_2_), and blood pressure (BP) will be recorded during surgery. All surgeons participating in this study have a surgical experience with over 200 lung cancer cases annually. Patients will receive mechanical ventilation with a tidal volume (VT) of 6–8 mL/kg of predicted body weight and a positive end-expiratory pressure (PEEP) of 6–8 cm of water. The fluid infusion speed and volume will be controlled according to urine output, and blood transfusion will be given when necessary. Intraoperative explorations include the examination for pleural effusion, lung fissure, adhesion, tumor invasiveness, etc. The surgery type will be restricted to lobectomy and the choice of thoracotomy or thoracoscopic surgery will be made by the surgeon-in-charge. One 28F chest tube (Yangzhou Hanjiang Huafei Medical Device Factory, Co., Ltd., Hanzhou, China) will be placed in the thoracic cavity from the 7th intercostal space to drain the excessive air and fluid. All patients will be applied the same conventional water-sealed drainage bottle without negative pressure. During the surgery, if the surgeon decides that a patient is no longer a candidate for lobectomy, alternative therapy will be provided, and the patient will be withdrawn from the trial, and a complete report will be made.

#### Study dropouts

All enrolled participants have the right to withdraw from the study for any reason at any time without any negative consequence for current or future treatment. All investigators have the right to terminate the participation of any subject at any time if deemed to be in the participant’s best interest. All reasons and circumstances of subject termination or withdrawal will be recorded timely in the case report form (CRF). Any participants who withdraw from this study will continue to be monitored as a part of this protocol, and efficacy and safety data will continue to be recorded. All data will be analyzed according to the intention-to-treat (ITT) principle.

#### Patient and public involvement

Patients and public are not involved in the trial design. The final results of this trial will be available to all the patients via letters, phone calls as well as email communication.

#### Statistical analysis

The statistical analysis will follow the ITT principle and a data analysis plan will be confirmed prior to the data being locked for final analysis, including the statistical methods, description of the missing data, and further quality control to guarantee the robustness of the findings reported. The main contents of the analysis are for the effectiveness and safety of the treatment groups. The analysis of all continuous variables will be presented as mean, standard deviation, median, quartile spacing, and maximum and minimum values. The analysis of all dichotomous variables will be presented as rate, constituent ratio, and hazard ratio. Statistical comparisons among five groups will be mainly conducted with one-way analysis of variance followed by post hoc Tukey’s tests, described data [26]. Dichotomous data will be compared with chi-squared tests. Univariate and multivariate logistic regression analysis will also be applied. The factors (*p* < 0.05) in univariate analysis will be used for multivariate analysis. Only when overall difference is significant, two-to-two comparison will be considered. All the data will be checked twice by two independent researchers to guarantee facticity. If dropout rate is higher than the expected 10%, multiple imputation will be used to avoid pitfalls involved with listwise deletion of cases that have missing values [27]. We will perform explanatory subgroup analysis based on age, gender, comorbidities, surgery type, and so on. The difference between groups will be considered statistically significant if *p* < 0.05. No interim analyses are planned. Analysis will only be conducted after enrolment close. All data will be analyzed using SPSS (software version 25.0, Chicago, IL).

### Study organization

#### Data collection and management

JZ and WC designed the patient-reported outcomes (PRO) content of the trial protocol, and are responsible for PRO contents. All patients’ demographic and clinical data will be recorded by investigators independently. Preoperative data will be collected within 3 days after recruitment. Surgery data will be collected within 2 days after the operation. Postoperative data will be collected within 3 days after discharge. For patients discharged home, we will retrieve follow-up information by phone calls, and these data will be recorded within 3 days. The organizational structure of the trial is as follows. The steering committee, which comprises JZ, LL, and administrators at the West China Hospital, has full oversight over the design of the trial. We have a data management safety committee (DMSC) comprised of 5 independent investigators. They will supervise the study protocol adherence and confirm that the CRF is correctly completed and consistent with the original data. If there are any errors or omissions, the investigator will correct them immediately. The raw data will be marked clearly when revising and signed by the investigator with date when the modifications are made. All the data can only be obtained by the study researchers who have signed the confidential disclosure agreement. We do not plan to collect personal information about potential and enrolled participants beyond what is collected during normal hospitalization. To maintain confidentiality, electronic health information will be encrypted as per hospital protocol. After the trial, personally identifiable information will be omitted and placed in a separate database before any data analysis will be performed. The adherence to the study protocol, data collection, statistical analysis, and related safety issues will be strictly monitored by the Institutional Ethics Committee of West China Hospital, Sichuan University.

#### Adverse effects

Drug adverse effects including nausea, vomiting, dizziness, fatigue, drowsiness, pruritus, abdominal distention, bloating, constipation, euphoria, respiratory depression, and indigestion will be recorded timely. Medical treatment will be applied as necessary. Unexpected severe drug adverse effects will be reported to the ethics committee.

#### Primary and secondary outcomes

The primary outcome is total consumption of sufentanil in PCIA within the first 24 postoperative hours. PCIA sufentanil consumption will be measured automatically by the PCIA pump, and recorded by nurses trained for this trial. The secondary outcomes include (1) the NRS at the 6th, 12th, 24th, 48th, and 72th postoperative hours, and on the 1st, 3rd, and 6th postoperative months at rest and during coughing; data collection timepoints are pre-specified. The NRS, ranging from 0 (no pain) to 10 (worst pain imaginable), will be utilized to assess the severity of postoperative pain for all participants. The NRS is a patient-reported subjective scale, with evidence of its use and interpretation shown elsewhere [28]. Only the Chinese language version of the NRS question will be used. To minimize avoidable missing data, nurses are required to key in the results into an electronic chart directly after the patient encounter. NRS will be collected by an oral survey administrated by trained nurses to ask in a standardized manner, in the setting of the hospital ward, until discharge. At the 1st, 3rd, and 6th post-operative month, NRS will be asked with phone-call by the follow-up team; (2) the total consumption of sufentanil in PCIA within the first 6, 12, 48, 72 postoperative hours, and the total consumption of sufentanil in “rescue” PCIA; (3) incidence of treatment-related adverse effects: nausea, vomiting, dizziness, fatigue, drowsiness, pruritus, abdominal distention, bloating, constipation, euphoria, respiratory depression, and indigestion, and any adverse events observed by a nurse will also be assessed by an on-site investigator; (4) the cost of analgesia, time to chest tube removal, length of hospital stay, time to pass first flatus; and (5) the serum level of interleukin-1β (IL-1β), IL-6, IL-8, IL-2, tumor necrosis factor-α (TNF-α), C-reactive protein (CRP), prostaglandin E2 (PGE2), and 5-hydroxytryptamine (5-HT). Collection of blood samples for cytokine studies would be performed by trained nurses. The laboratory evaluation would be conducted by technicians in the Department of Laboratory Medicine, blinded to treatment groups. Laboratory results will be placed in the electronic chart. Specimens will be destroyed and not stored for ancillary studies.

#### Adverse event

In this study, the adverse event is defined as any untoward treatment-related medical occurrence, including any treatment-related unfavorable and unintended sign, symptom, or disease. Within 30 days after the last treatment of this protocol, any newly occurring events or previous conditions (level of postoperative pain) increased in intensity or frequency would be considered as the adverse event. Lack of efficacy before the study end would not be considered an adverse event. The adverse event would be evaluated and reported to the principal investigator according to Common Terminology Criteria for Adverse Events (v4.03) [29].

#### Auditing

The ethics committee will conduct local monitoring of trial quality after the first patients have been enrolled. The trial database provides on-going data quality checking, including the recruitment rate, data quality, and adverse event reporting.

## Discussion

Sufficient postoperative pain control is essential to surgical outcomes and postoperative recovery [30]. Dezocine and flurbiprofen axetil, representatives of opioids and NSAIDs, respectively, are important components of perioperative pain management [20, 31, 32], and the combined application of the two differentially acting drugs is becoming widely accepted. Our center generally adopted a combination of PCIA sufentanil with intravenous flurbiprofen or parecoxib sodium. Oral opioids are only used when needed. Adverse effects of dezocine, including nausea, vomiting, respiratory depression, and opioid withdrawal, are frequently reported and can be life-threatening in severe cases. Likewise, flurbiprofen axetil use can be complicated by nausea, vomiting, diarrhea, and perforation of gastrointestinal tracts. Nevertheless, our study indicated a minimization of adverse effects with maximization of analgesic effects by incorporating flurbiprofen axetil to dezocine. A paucity of data investigating the synergistic antinociceptive effect and the resultant decrease of adverse effects left the optimal regimen undefined. To fill this gap of knowledge, we will conduct this clinical trial to find the optimal analgesic regimen for the pain management after lobectomy. This study will investigate the pain score-NRS, the incidence of drug-related adverse effects, and the precise total consumption of sufentanil in PCIA.

Several limitations of this trial should be noted. First, the characteristics of single-center trial, along with the limited availability of dezocine, will limit the generalizability of the study. A multiple-center large-sample clinical trial may be warranted in the future. Second, a diverse group of participants may be enrolled which could potentially affect the final results. Third, individual threshold of pain perception and the duration of surgery demanded by individual conditions may have an impact on the severity of postoperative pain. Thus, we should cautiously interpret our results.

To our knowledge, this study will be the first RCT on the analgesic effects and drug-related adverse events of combined dezocine and flurbiprofen axetil after lobectomy. This study may contribute to decision-making when an individualized pain control is requested and achieve the optimal regimen with the least complications and the most satisfactory.

### Trial status

Enrolment is not begun. Participant recruitment start date is planned to be June 1, 2020. The approximate recruitment end date is May 31, 2021. The protocol version is Version 2.0, dated August 24, 2018.

## Supplementary Information

**Additional file 1.** SPIRIT 2013 checklist: recommended items to address in a clinical trial protocol and related documents.

## Data Availability

Results of this study are going to be published in a peer-reviewed journal. There are plans to publish the full statistical code in the final manuscript of the RCT after the trial has ended. There are no concrete plans to publish a participant-level dataset at this time.

## Abbreviations

PCIA: Patient-controlled intravenous analgesia
NRS: Numeric rating scales
NSAIDs: Non-steroidal anti-inflammatory drugs
RCT: Randomized controlled trial
ECG: Electrocardiogram
SpO2: Pulse oxygen saturation
BIS: Bispectral index
EtCO2: End-tidal carbon dioxide tension
BP: Blood pressure
VT: Tidal volume
PEEP: Positive end-expiratory pressure
CRF: Case report form
ITT: Intention-to-treat
PRO: Patient-reported outcomes
DMSC: Data management safety committee
IL-: Interleukin-
TNF-α: tumor necrosis factor-α
CRP: C-reactive protein
PGE2: Prostaglandin E2
5-HT: 5-hydroxytryptamine
